# Supplementary material for: Background selection and biased gene conversion affect more than 95% of the human genome and bias demographic inferences
Source: eLife. 2018 Aug 23;7:e36317. doi: 10.7554/eLife.36317 (PMC6177262; doi:10.7554/eLife.36317)
Supplement: Transparent reporting form [file elife-36317-transrepform.docx]

***eLife’s* transparent reporting form**

We encourage authors to provide detailed information *within their submission* to facilitate the interpretation and replication of experiments. Authors can upload supporting documentation to indicate the use of appropriate reporting guidelines for health-related research (see [EQUATOR Network](http://www.equator-network.org/%20)), life science research (see the [BioSharing Information Resource](https://biosharing.org/)), or the [ARRIVE guidelines](http://www.plosbiology.org/article/info:doi/10.1371/journal.pbio.1000412) for reporting work involving animal research. Where applicable, authors should refer to any relevant reporting standards documents in this form.

If you have any questions, please consult our Journal Policies and/or contact us: [editorial@elifesciences.org](mailto:editorial@elifesciences.org).

**Sample-size estimation**

- You should state whether an appropriate sample size was computed when the study was being designed
- You should state the statistical method of sample size computation and any required assumptions
- If no explicit power analysis was used, you should describe how you decided what sample (replicate) size (number) to use

Please outline where this information can be found within the submission (e.g., sections or figure legends), or explain why this information doesn’t apply to your submission:

*This information doesn’t apply to our submission as we use already published data (i.e. annotated SNPs from humans). Filtering steps are described in the method section (Data processing and annotations, line 410-437, page 19-20)*

**Replicates**

- You should report how often each experiment was performed
- You should include a definition of biological versus technical replication
- The data obtained should be provided and sufficient information should be provided to indicate the number of independent biological and/or technical replicates
- If you encountered any outliers, you should describe how these were handled
- Criteria for exclusion/inclusion of data should be clearly stated
- High-throughput sequence data should be uploaded before submission, with a private link for reviewers provided (these are available from both GEO and ArrayExpress)

Please outline where this information can be found within the submission (e.g., sections or figure legends), or explain why this information doesn’t apply to your submission:

*This information doesn’t apply to our submission as we obtained the data from already published dataset (identification of samples and corresponding citation are provided in the manuscript, see table S1).*

**Statistical reporting**

- Statistical analysis methods should be described and justified
- Raw data should be presented in figures whenever informative to do so (typically when N per group is less than 10)
- For each experiment, you should identify the statistical tests used, exact values of N, definitions of center, methods of multiple test correction, and dispersion and precision measures (e.g., mean, median, SD, SEM, confidence intervals; and, for the major substantive results, a measure of effect size (e.g., Pearson's r, Cohen's d)
- Report exact p-values wherever possible alongside the summary statistics and 95% confidence intervals. These should be reported for all key questions and not only when the p-value is less than 0.05.

Please outline where this information can be found within the submission (e.g., sections or figure legends), or explain why this information doesn’t apply to your submission:

*For any confidence interval estimates we described the method (block-bootstrap etc.) in the legend of the figures and in the material and methods section (paragraphs “Site frequency spectrum” lines 499-503 + “Block bootstrap procedure” lines 505-510 + “demographic inferences” lines 520-525 + “individual based simulations” lines 551-554).*

(For large datasets, or papers with a very large number of statistical tests, you may upload a single table file with tests, Ns, etc., with reference to sections in the manuscript.)

**Group allocation**

- Indicate how samples were allocated into experimental groups (in the case of clinical studies, please specify allocation to treatment method); if randomization was used, please also state if restricted randomization was applied
- Indicate if masking was used during group allocation, data collection and/or data analysis

Please outline where this information can be found within the submission (e.g., sections or figure legends), or explain why this information doesn’t apply to your submission:

*This information does not apply to our submission as we performed population genetics analyses and no clinical studies. We grouped individuals per populations and sub-group, as described in 1000G or SGDP project.*

**Additional data files (“source data”)**

- We encourage you to upload relevant additional data files, such as numerical data that are represented as a graph in a figure, or as a summary table
- Where provided, these should be in the most useful format, and they can be uploaded as “Source data” files linked to a main figure or table
- Include model definition files including the full list of parameters used
- Include code used for data analysis (e.g., R, MatLab)
- Avoid stating that data files are “available upon request”

Please indicate the figures or tables for which source data files have been provided:

*We provide the setting files for demographic inferences, a summary table of the genotype table characteristics and source code to reproduce the figures here:* [*http://datadryad.org/review?doi=doi:10.5061/dryad.t76fk80*](http://datadryad.org/review?doi=doi:10.5061/dryad.t76fk80)

*Setting parameters for individual based simulations are described in the method sections.*
